# Supplementary figures and images for: Pharmacological Inhibition of Microglial Proliferation Supports Blood–Brain Barrier Integrity in Experimental Autoimmune Encephalomyelitis
Source: Cells. 2025 Mar 12;14(6):414. doi: 10.3390/cells14060414 (PMC11941641; doi:10.3390/cells14060414)

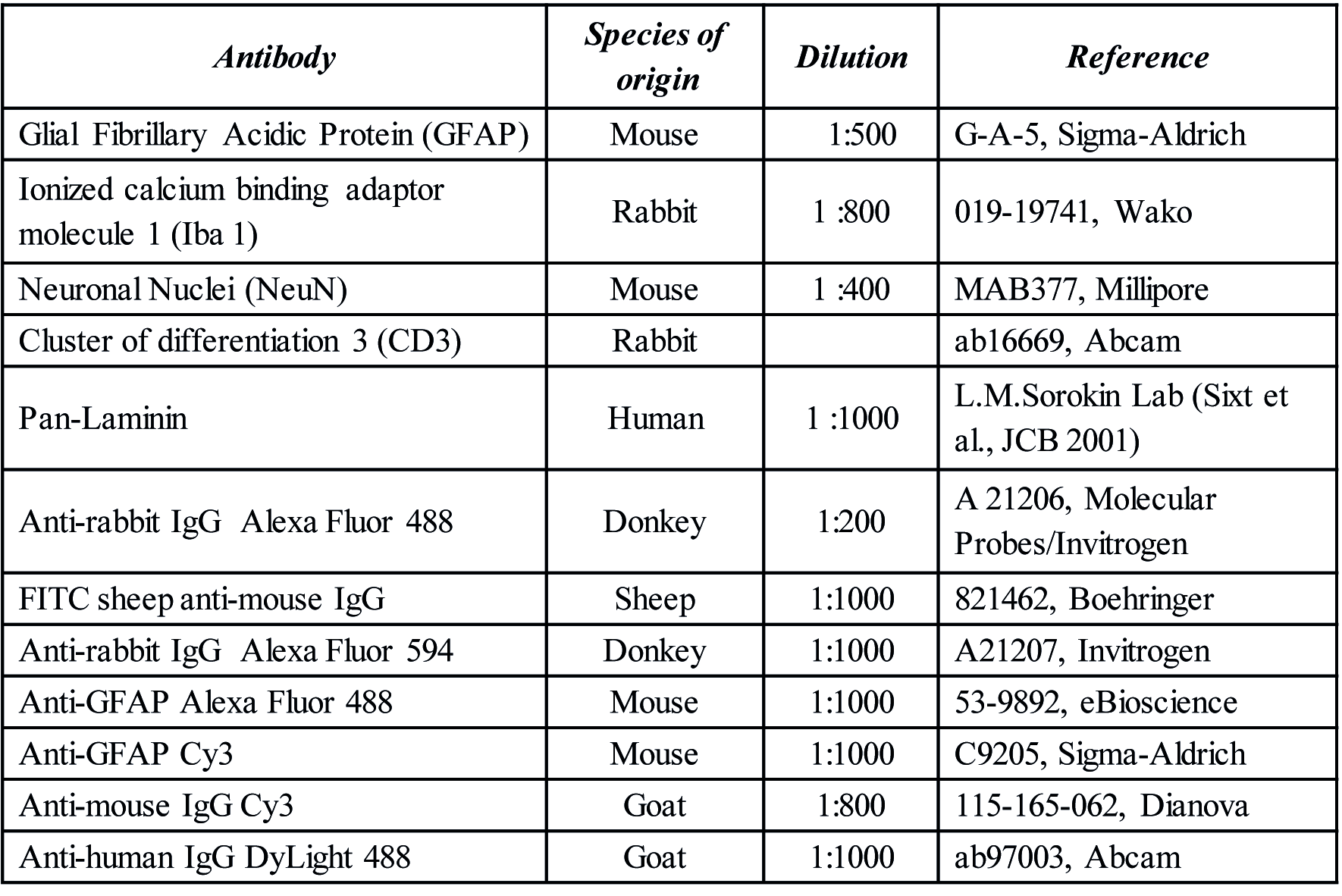

Supplement: Supplementary file 1 [file cells-14-00414-s001.zip › supplementary Table 1.tif]

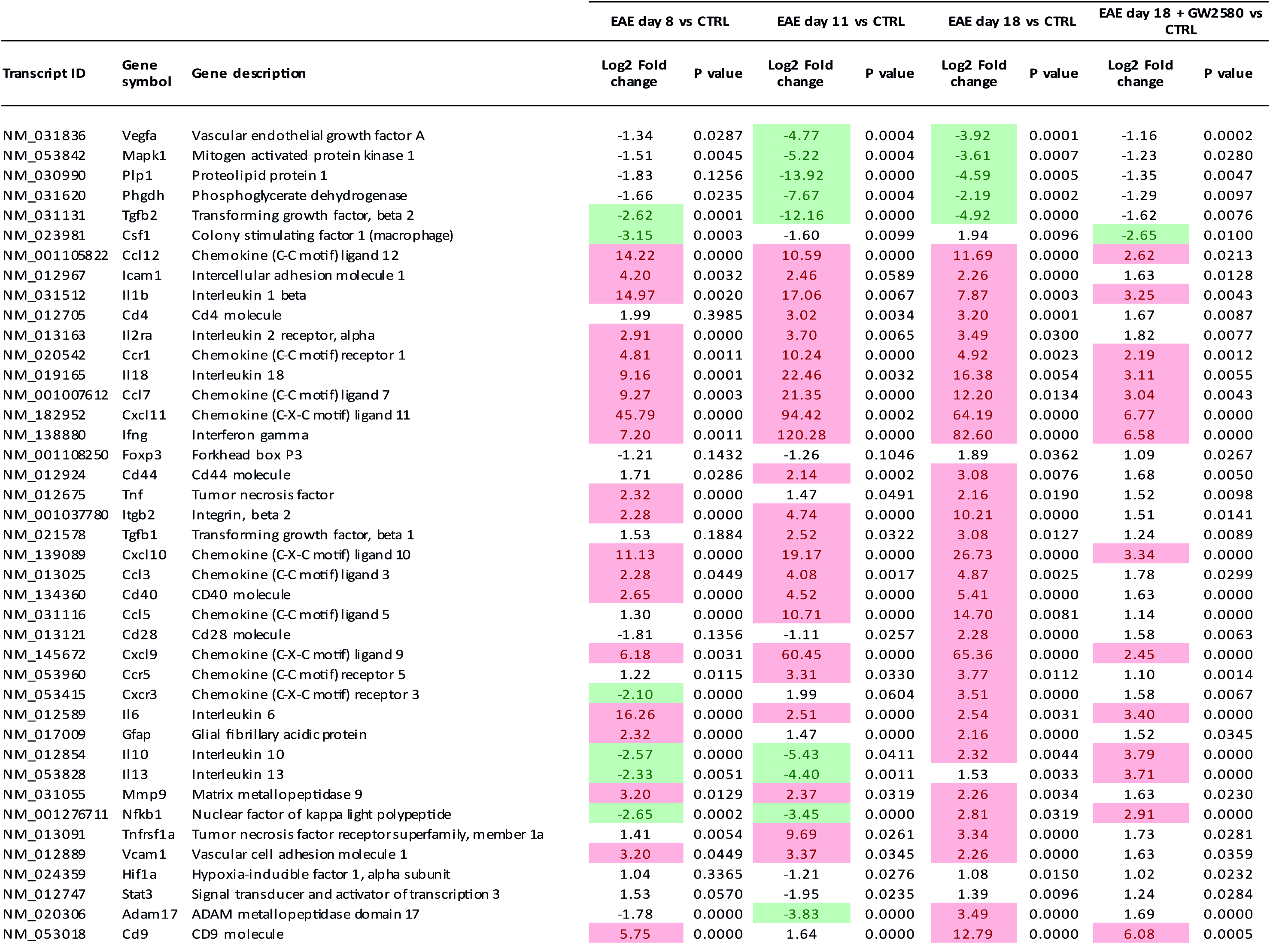

Supplement: Supplementary file 1 [file cells-14-00414-s001.zip › supplementary Table 2.tif]
